# Pericentromeric repetitive ncRNA regulates chromatin interaction and inflammatory gene expression

**DOI:** 10.1080/19491034.2022.2034269

**Published:** 2022-02-15

**Authors:** Kenichi Miyata, Akiko Takahashi

**Affiliations:** a Project for Cellular Senescence, Cancer Institute, Japanese Foundation for Cancer Research, Tokyo, Japan; b Cancer Cell Communication Project, NEXT-Ganken Program, Japanese Foundation for Cancer Research, Tokyo, Japan; c Advanced Research & Development Programs for Medical Innovation (PRIME), Japan Agency for Medical Research and Development (Amed), Tokyo, Japan

**Keywords:** Cellular senescence, CTCF, pericentromeric RNA, senescence-associated secretory phenotype, small extracellular vesicles

## Abstract

Cellular senescence provokes a dramatic alteration of chromatin organization and gene expression profile of proinflammatory factors, thereby contributing to various age-related pathologies via the senescence-associated secretory phenotype (SASP). Chromatin organization and global gene expression are maintained through the CCCTC-binding factor (CTCF). However, the molecular mechanism underlying CTCF regulation and its association with SASP gene expression remains to be fully elucidated. A recent study by our team showed that noncoding RNA (ncRNA) derived from normally silenced pericentromeric repetitive sequences directly impair the DNA binding of CTCF. This CTCF disturbance increases the accessibility of chromatin at the loci of SASP genes and caused the transcription of inflammatory factors. This mechanism may promote malignant transformation.

Cellular senescence is a state of irreversible cell cycle arrest induced by many stressors, i.e., aging, obesity, radiation, and chemotherapy [1]. Senescent cells that accumulate *in vivo* during aging communicate with surrounding tissues through proinflammatory protein production, termed the senescence-associated secretory phenotype (SASP), which plays several physiological and pathological roles. In aged individuals, inflammatory SASP factors promote numerous age-related diseases, including some cancers [2–7]. Therefore, elucidating the regulatory mechanism of SASP is vital for developing new preventive and therapeutic strategies against age-related cancer. Recent studies have shown that abnormal nuclear morphologies, observed as micronuclei or nuclear buds, induce SASP gene expression through the activation of the DNA sensing pathway during cellular senescence [8–12]. Additionally, Criscione *et al*. reported that cellular senescence causes a dramatic alteration of chromatin organization [13]; however, how chromatin organization dramatically change in senescent cells is not ultimately understood. Here, it was disclosed that the functional impairment of CTCF by pericentromeric repetitive ncRNA results in an alteration of chromatin interaction, followed by the upregulation of SASP-like inflammatory genes accelerating malignant transformation [14].

First, the authors have hypothesized that an aberrant chromatin architecture observed in senescent cells might be associated with SASP and have conducted the analysis of genome-wide chromatin accessibility (assay for transposase-accessible chromatin sequencing; ATAC-seq) followed by gene expression (RNA-Seq) by comparing the proliferating and X-ray-induced senescent human diploid fibroblasts (IMR-90). The combinational analysis showed that the loci containing pericentromeric repetitive sequences called human satellite II (hSATII), which is epigenetically silenced in normal somatic cells, were highly accessible, and hSATII ncRNA expression were markedly upregulated in senescent IMR-90 cells compared with proliferating cells. Conversely, it has been reported that hSATII ncRNA expression is upregulated in senescent cells and many types of cancer [15–22]; the function of the ncRNA in SASP regulation remains to be elucidated.

Accumulating evidence uncovers that diverse noncoding RNAs (ncRNAs) are involved in various biological processes. Especially, a subset of ncRNAs epigenetically regulates gene expression through chromatin remodeling. Li *et al*. reported that enhancer RNA transcripts up on 17β-estradiol (E2) could be functionally essential for the actions of E2-regulated gene enhancers, at least in part by contributing to the dynamic generation or stabilization of enhancer-promoter looping in human breast cancer cells [23,24]. Interestingly, Camacho *et al*. reported that murine pericentromeric satellite RNA *in cis* stabilizes heterochromatin retention of Suv39h [25]. Additionally, Mallm *et al*. reported that murine centromeric satellite RNA *in trans* regulates telomerase activity in embryonic stem cells [26]. These reports propose that human pericentromeric satellite (hSATII) ncRNA can also affect gene expression and/or chromatin organization in senescent cells. To unravel the biological effects of hSATII ncRNA expression, the authors overexpressed hSATII ncRNA in conditionally immortalized human fibroblasts [27]. The ectopic expression of hSATII ncRNA induced SASP-like inflammatory gene expression and altered the chromatin accessibility of the loci of SASP genes [28]. These effects were not observed using ectopic expression of centromeric human satellite alpha (hSATα) ncRNA. These data suggest that hSATII ncRNA regulates SASP-like inflammatory gene expression by changing the chromatin accessibility during cellular senescence.

To uncover the molecular mechanism of how hSATII ncRNA promotes SASP-like inflammatory gene expression, the authors attempted to identify hSATII ncRNA-binding proteins. RNA pull-down followed by using mass spectrometry analysis identified CCCTC-binding factor (CTCF) as a hSATII ncRNA-binding protein, and RNA immunoprecipitation analysis showed that the zinc finger (ZF) DNA- and RNA-binding domains of CTCF are essential for their binding to hSATII ncRNA. Significantly, the upregulation of SASP-like inflammatory gene expression induced by hSATII ncRNA was canceled in cells expressing excessive CTCF. Conversely, CTCF depletion caused SASP-like inflammatory gene expression in proliferating cells. Interestingly, the authors found that CTCF depletion increases the expression of not only SASP-like inflammatory genes but also hSATII RNA. Moreover, the expression of CTCF decreases during cellular senescence. Together, although it is not clear whether CTCF is the direct or indirect trigger for hSATII RNA expression, these findings imply that the loss of CTCF drives hSATII RNA expression during cellular senescence. Furthermore, the authors investigated the expression of mouse major satellite (MajSAT) RNA, which is located at the pericentromeric locus of chromosomes, as well as human hSATII ncRNA. The expression of MajSAT RNA also increased in senescent mouse embryonic fibroblasts. Additionally, MajSAT RNA, but not mouse centromeric minor satellite (MinSAT) RNA, bound to CTCF, leading to upregulation of SASP-like inflammatory genes. Altogether, these findings indicate that the functional impairment of CTCF by pericentromeric satellite ncRNAs induces the expression of SASP-like inflammatory genes during cellular senescence.

Because the DNA-binding domain of CTCF is essential for the maintenance of genomic integrity and was relevant to its binding to hSATII ncRNA, the authors hypothesized that hSATII ncRNA disturbs the DNA-binding capacity of CTCF through direct binding to its ZF domains. Chromatin immunoprecipitation (ChIP)-seq analysis showed that ectopic expression of hSATII ncRNA altered the distribution of CTCF. Remarkably, ChIP-qPCR and electrophoretic mobility shift assay showed that hSATII ncRNA inhibited the DNA-binding capacity of CTCF to an imprinting control region, a representative CTCF binding site [29]. Moreover, chromosome conformation capture (3C) assay in the vicinity of the loci of SASP genes elucidated that the ectopic expression of hSATII ncRNA significantly weakened chromatin interactions in that region, followed by the upregulation of SASP-like inflammatory gene expression. Together, these data indicate that the upregulation of hSATII ncRNA induces a conformational change of chromatin structure in some SASP gene loci (Figure 1).

**Figure 1.**
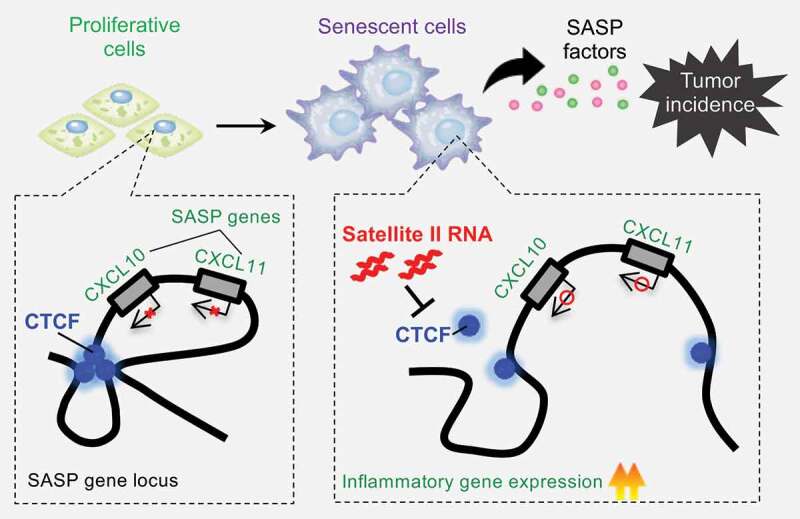
The noncoding RNA (ncRNA) transcribed from the pericentromeric repetitive satellite sequences changes the distribution of CCCTC-binding factor (CTCF) binding on the genome, thereby inducing senescence-associated secretory phenotype (SASP)-like inflammatory gene expression through the functional impairment of CTCF in senescent cells. Additionally, pericentromeric satellite RNA provokes tumorigenesis in a cell-autonomous or non-cell-autonomous manner through a pathway involving exosomes, a type of small extracellular vesicle (EV). This is a novel mechanism of CTCF regulation by pericentromeric satellite RNA during cellular senescence, which may contribute to the risk of tumorigenesis.

Cellular senescence causes a dramatic alteration of chromatin organization [13,30]; however, its effect on gene expression and implications for senescent cells is not fully understood. The authors identified ncRNA derived from pericentromeric repetitive elements as a novel inducer of SASP-like inflammatory gene expression that alters chromatin interaction. Normally, the pericentromeric satellite RNA expression is extremely low in normal cells [15]. Therefore, the authors considered that it is insufficient for RNA to disturb CTCF function under physiological conditions, but not in senescent and tumor cells that aberrantly express pericentromeric satellite RNA. In a previous study, Zirkel *et al*. showed that nuclear depletion of the high-mobility group B protein (HMGB2) provokes the alteration of CTCF distribution during cellular senescence [30]. Moreover, Lehman *et al*. reported that different stressors alter CTCF-RNA interaction [31]. These reports support the authors’ findings that pericentromeric satellite RNA upregulated during cellular senescence directly binds to CTCF and disturbs CTCF function. Alternatively, some reports show that CTCF-mediated promoter–enhancer interaction is a critical regulator of gene expression [32]. However, decreased CTCF binding to DNA is associated with a loss of insulation between the topological domains and aberrant gene activation in various tumors such as T cell acute lymphoblastic leukemia (T-ALL), gliomas, and gastrointestinal stromal tumors [33–35]. Therefore, the authors speculate that hSATII ncRNA alters chromosomal organization by directly impairing the binding of CTCF to DNA, thereby causing the formation of a novel promoter–enhancer interaction, which may be a cause of aberrant expression of SASP-like inflammatory genes.

Small extracellular vesicles (EVs) secreted from cancer and stromal cells dynamically contribute to tumor incidence and progression in a non–cell-autonomous manner in the tumor microenvironment [36–41]. Intriguingly, the amounts of hSATII ncRNA were higher in small EVs derived from senescent cells than in those derived from proliferating cells. Thus, the authors’ data suggest that hSATII ncRNA derived from senescent stromal cells are transferred into surrounding cells through small EVs and function as a SASP-like inflammatory factor in the tumor microenvironment. Further, the authors found that hSATII ncRNA was highly detectable in cancer cells in surgical specimens from patients with primary colon carcinoma. Strikingly, the population of hSATII ncRNA-positive cells was significantly higher among cancer-associated fibroblasts than fibroblasts in normal stromal tissues.

These findings highlight the new role of the pericentromeric satellite RNA, which supports tumor development in a non–cell-autonomous manner through the secretion of SASP-like inflammatory factors and small EVs. The SASP factors are thought to promote multiple age-related diseases including some cancers, such as breast, liver and colon cancers. However, blockage of all SASP factors *in vivo* could be toxicity because SASP factors play important roles in various physiological processes [7]. In addition, some SASP factors are also important for cancer prevention by immune cells at the early stages of cancer development [42], suggesting that it is a key to alter only harmful SASP factors involved in tumor incidence and/or progression to cure for human cancer. hSATII RNA transcribed in senescent and various cancer cells but not in normal cells is a strong driver to provoke SASP-like inflammatory gene expression. Therefore, the authors think that inhibition of hSATII transcripts, e.g., nucleic acid therapeutic, could lead to a cure for human cancer safely. Understanding this molecular mechanism can facilitate the development of novel preventive and therapeutic strategies against age-related pathologies in the future.
